# Minimally invasive strabismus surgery for horizontal rectus muscle reoperations

**DOI:** 10.1136/bjo.2008.145110

**Published:** 2008-09-09

**Authors:** D S Mojon

## Abstract

**Aims:**

To study if minimally invasive strabismus surgery (MISS) is suitable for rectus muscle reoperations.

**Methods:**

The study presents a series of consecutive patients operated on by the same surgeon at Kantonsspital St Gallen, Switzerland with a novel MISS rectus muscle reoperation technique. Surgery is done by applying two small radial cuts along the muscle insertion. Through the tunnel obtained after muscle separation from surrounding tissue, a recession, advancement or plication is performed.

**Results:**

In 62 eyes of 51 patients (age 35.4 (SD 16.3) years) a total of 86 horizontal rectus muscles were reoperated. On the average, the patients had 2.1 strabismus surgeries previously. Preoperative logMAR visual acuity was 0.38 (0.82) compared with 0.37 (0.83) at 6 months (p>0.1). On the first postoperative day, in the primary gaze position conjunctival and lid swelling and redness was hardly visible in 11 eyes, discrete in 15 eyes, moderate in 11 eyes and severe in 15 eyes. One corneal dellen and one corneal erosion occurred, which both quickly resolved. The preoperative deviation at distance for esodeviations (n = 15) of 12.5 (8.5)° decreased to 2.6 (7.8)° at 6 months (p<0.001). For near, a decrease from 12.0 (10.1)° to 2.9 (1.6)° was observed (p<0.001). The preoperative deviation at distance for exodeviations (n = 35) of −16.4 (8.5)° decreased to −7.9 (6.5)° at 6 months (p<0.005). For near, a decrease from −16.5 (11.4)° to −2.9 (1.5)° was observed (p<0.005). Within the first 6 months, only one patient had a reoperation. At month 6, in four patients a reoperation was planned or suggested by us because of unsatisfactory alignment. No patient experienced persistent diplopia or necessitated a reoperation because of double vision. Stereovision improved at month 6 compared with preoperatively (p<0.01).

**Conclusions:**

The study demonstrates that a small-cut, minimal dissection technique allows to perform rectus muscle reoperations. The MISS technique seems to reduce conjunctival and lid swelling in the direct postoperative period.

Minimally invasive surgical procedures reduce tissue traumatism, postoperative patient dis-comfort, hospital stay, working disability, and the economic impact of surgery.^1^ ^2^ They are now routine in many fields of surgery. In ophthalmology, the following minimally invasive procedures are in use: phacoemulsification for cataracts,^3^ non-penetrating techniques^4^ and miniature drainage implants^5^ for glaucoma, transconjuctival approaches^6^ and minimal buckling^7^ for vitreoretinal surgery, endoscopic techniques for the lacrimal system,^8^ and small-incisions for lids.^9^ In strabismus surgery, a smaller conjunctival incision increases the postoperative quality of life, cosmesis, and the function of the operated muscle. The opening size also influences the ease to perform revision surgery. The majority of surgeons use the limbal approach first described by Harms in 1949^10^ and later popularised by von Noorden.^11^ This approach allows one to easily perform primary or revision surgery in horizontal rectus muscles^11^ ^12^ (fig 1A,B). Several other conjunctival openings have been proposed by these authors: Swan and Talbott,^13^ Parks,^14^ Velez^15^ and Santiago *et al*.^16^ In a previous study, I described a novel minimally invasive strabismus surgery (MISS) technique for rectus muscle recessions and plications and compared it with the usual limbal opening.^17^ The MISS operation is performed by applying two small radial cuts along the muscle insertion. After the muscle is separated from its surrounding tissue, a recession or a plication is done through the resulting tunnel. MISS patients had better visual acuities and less lid swelling the day after surgery, indicating that the technique is superior in the direct postoperative period. A conjunctival opening situated at a reasonable distance from the limbus might decrease the incidence of corneal dellen formation and avoid a prolapse of the Tenon capsule. There is also evidence that non-limbal strabismus surgery affects less perilimbal blood supply and may safeguard anterior segment ischaemia in high-risk patients.^18^

**Figure 1:**
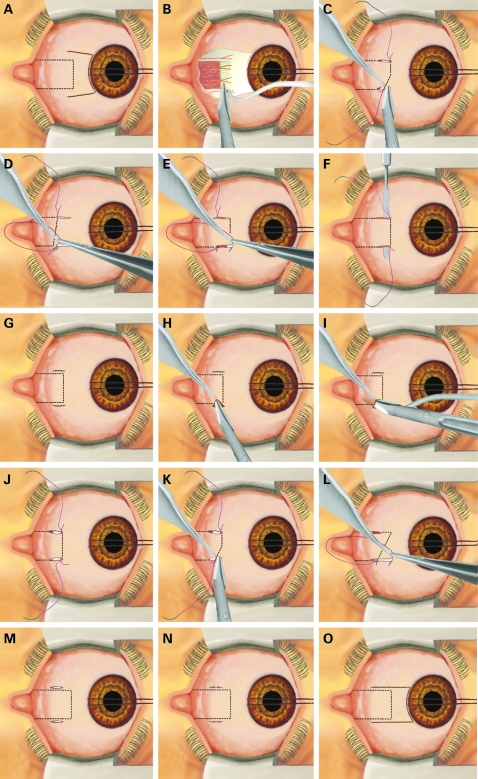
Schematic representation of Harms conjunctival opening and the surgical technique for MISS rectus muscle reoperations: (A, B) Harms limbal opening with two relaxing radial cuts. Recession: (C) A limbal traction suture is applied to rotate the eyeball away from the field of surgery. Two small radial cuts are performed, one along the superior and one along the inferior muscle margin. With blunt Wescott scissors using the two cuts for access, the episcleral tissue is separated from the muscle sheath and the sclera, and the muscle is hooked. A meticulous dissection of the check ligaments and intramuscular membrane is performed. Two sutures are applied to the superior and inferior border of the muscle tendon as close as possible to the insertion. The tendon is detached using scissors. (D) After measurement of the amount of recession, the tendon is reattached with the two sutures to the sclera. (M, N) The surgical procedure is finished by applying two sutures to each of the two small cuts. Plication: (E) After applying a limbal traction suture and performing the two small cuts, two sutures are applied to the upper and lower borders of the muscle at the distance from the tendon insertion site corresponding to the plication amount. The sutures are passed at the superior and inferior tendon insertions respectively. (F) An iris spatula is inserted between the tendon and the sutures and the muscle is plicated. (M, N) The surgical procedure ends by applying two sutures to each of the two small cuts. Advancement: (G) After applying a limbal traction suture, the two small radial openings are created. The anterior margins of the cuts are at the level of the actual tendon insertion. (H) With blunt Wescott scissors using the two cuts for access, the episcleral tissue is separated from the muscle sheath and the sclera. (I) The muscle is hooked. A meticulous dissection of the check ligaments and intramuscular membrane is performed. (J) Two sutures are applied to the superior and inferior border of the muscle tendon as close as possible to the insertion. (K) Then, the tendon is detached using scissors. (L) After measurement of the amount of advancement, the tendon is reattached with the two sutures to the sclera. (M, N) The surgical procedure is finished by applying two sutures to each of the two small cuts. (O) If a better visualisation of the operating site becomes necessary, the two cuts can be prolonged and joined at the limbus.

This study describes how the previously published MISS technique for primary rectus muscle surgery^17^ can be adapted to perform rectus muscle reoperations with minimal anatomical disruption and presents the results of the first series of patients.

## PATIENTS

This study reports the results of the first 51 consecutive patients operated on with a MISS reoperation technique at Kantonsspital St Gallen. The investigation followed the tenets of the Declaration of Helsinki. The president of the Ethical Committee of Kanton St Gallen has approved the use of this new technique.

### Patients undergoing MISS horizontal reoperations

Inclusion criteria: All consecutive patients needing horizontal rectus muscle reoperations by the author between May 2003 and June 2007 were included. Exclusion criteria: Patients with excessive conjunctival scarring from previous surgery necessitating simultaneous conjunctival grafting, need for retroequatorial fixation sutures or muscle transpositions, simultaneous vertical rectus or obliquus muscle surgery, or strongly restricted passive motility. All patients had at least one complete orthoptic examination 5 days before the surgical procedure, on the first postoperative day and after 6 months (range 5–7 months) at the Department of Strabismology and Neuro-Ophthalmology, Kantonsspital, St Gallen, Switzerland. Between day 1 and month 6, only patients harbouring a complication or not referred by an ophthalmologist were seen at our department. The referring ophthalmologist followed the other patients. The schedule of follow-up visits in between was at day 10 (range 1–2 weeks) and week 4 (range 3–5 weeks).

### Outcome measures

The following parameters were registered: final alignment, binocular single vision, variations in vision, refraction, and number and types of complications and retreatments required during the first 6 months after surgery or at the 6 months postoperative visit. In patients with central fixation squint angles were always measured with the alternating cover test. Otherwise, angles were determined by centralising the corneal reflex using prisms in front of the fixating eye (Krimsky test). On the first postoperative day, conjunctival and lid swelling and redness were determined in primary gaze position, when the cuts were covered by the eyelids. In eight eyes (12.9%) the quality of the slit-lamp photographs or the chart documentation was insufficient to classify the lid and conjunctival status. None of these eight patients had a conjunctival or lid abnormality at month 6. The following ordinal scale was used: redness and swelling of eyelid and conjunctiva not visible from 1 m = “hardly visible”; ptosis of not more than 1 mm and only minimal redness or swelling of conjunctiva visible from 1 m = “discrete”; immediate visibility of redness from 1 m or ptosis of more than 1 mm = “moderate”; conjunctival chemosis or subconjuctival haemorrhage, ptosis of more than 3 mm or lid haemorrhage = “severe.”

## METHODS

### Principle of revision surgery

If the actual deviation was secondary to a previous recession, a muscle advancement of the recessed muscle was always performed. Otherwise muscle reinforcements were performed by plications. All horizontal muscle procedures were combined; a recession was combined either with an ipsilateral placation or with an ipsilateral advancement. If necessary, usually in angles >25°, an additional recession was performed in the contralateral eye. Preoperatively, usually the planned intraoperative placement of the keywhole openings was determined using information about the type and amount of previous surgery. If not available, the site was established either preoperatively by location of the muscle insertion using the slit-lamp or intraoperatively by moving the eye using the traction suture. Apart from eyes with excessive scarring or with abundant Tenon, moving the eye frequently allows one to distinguish which vessels are conjunctival and which belong to the muscle, thus permitting the actual insertion site to be determined.

### Schematic representation of the surgical technique for MISS rectus muscle reoperations

Surgery is performed with the operating microscope under general anaesthesia. There is no need for an assistant, since all surgical steps can be performed alone.

#### Recession

The technique is similar to the technique described in the previous article about MISS.^17^ Therefore, only a brief description is given. First, a limbal traction suture (Silkam 6-0, B. Braun Medical, Switzerland) is applied to rotate the eyeball away from the field of surgery (fig 1C). At any time, direct contact of the traction suture with the cornea has to be avoided. Then, two small radial cuts are performed, one along the superior and one along the inferior muscle margin (fig 1C). The anterior margin of the cut is at the level of the actual tendon insertion. The size of the cuts should be 1 mm less than the amount of the planned muscle displacement. Using blunt Wescott scissors using the two cuts for access, the episcleral tissue is separated from the muscle sheath and the sclera. When the borders of the muscles have been identified, the muscle is hooked. Now, a meticulous dissection of the check ligaments and intramuscular membrane is performed 6–7 mm backward to the insertion. The resulting tunnel allows one to perform the recession. Two sutures (Vicryl 7-0, Ethicon, Switzerland) are applied to the superior and inferior border of the muscle tendon as close as possible to the insertion. Then, the tendon is detached using Wescott scissors (fig 1C). If necessary, haemostasis is performed. After measurement of the amount of recession, the tendon is reattached with the two sutures to the sclera (fig 1D). The surgical procedure is finished by applying two sutures (Vicryl Rapid 8-0, Ethicon, Switzerland) to each of the two small cuts (fig 1M,N).

#### Plication

The technique is similar to the technique already described in a previous MISS article.^17^ Therefore, only a brief description is given. After applying a limbal traction suture (Silkam 6-0, B. Braun Medical, Switzerland) to rotate the eyeball away from the field of surgery and performing the two small cuts, two sutures (Vicryl 7-0, Ethicon, Switzerland) are applied to the upper and lower borders of the muscle at the distance from the tendon insertion site corresponding to the plication amount (fig 1E). Then, the sutures are passed at the superior and inferior tendon insertions respectively (fig 1E). An iris spatula is inserted between the tendon and the sutures and the muscle is plicated (fig 1F). The surgical procedure ends by applying two sutures (Vicryl Rapid 8-0, Ethicon, Switzerland) to each of the two small cuts (fig 1M,N).

#### Advancement

After applying a limbal traction suture (Silkam 6-0, B. Braun Medical, Switzerland) to rotate the eyeball away from the field of surgery, two small radial cuts are performed, one along the superior and one along the inferior muscle margin (fig 1G). The anterior margin of the cut is at the level of the actual tendon insertion. With blunt Wescott scissors using the two cuts for access, the episcleral tissue is separated from the muscle sheath and the sclera (fig 1H). When the borders of the muscles have been identified, the muscle is hooked. Now, a meticulous dissection of the check ligaments and intramuscular membrane is performed 6–7 mm backward to the insertion. The resulting tunnel allows one to perform the advancement (fig 1I). Two sutures (Vicryl 7-0, Ethicon, Switzerland) are applied to the superior and inferior border of the muscle tendon as close as possible to the insertion (fig 1J). Then, the tendon is detached using Wescott scissors (fig 1K). If necessary, haemostasis is performed. After measurement of the amount of advancement, the tendon is reattached with the two sutures to the sclera (fig 1L). In order to perform the reattachment without enlarging the opening size, the cut has to be displaced anteriorly using a forceps. The surgical procedure is finished by applying two sutures (Vicryl Rapid 8-0, Ethicon, Switzerland) to each of the two small cuts (fig 1M,N). If a better visualisation of the operating site becomes necessary, the two cuts can be prolonged and joined at the limbus (fig 1O).

**Table 2:** Postoperative characteristics of minimally invasive strabismus surgery patients and controls

Characteristics	Values
Amount of surgery	
Recession	4.6 (1.8) mm
Plication	6.7 (1.6) mm
Advancement	6.4 (2.6) mm
LogMAR visual acuity	
Preoperative	0.38 (0.82)
Postoperative at month 6	0.37 (0.83)
Alignment at month 6	
Near and distance ⩽10 PD	23/51 (45%, CI 32 to 59%)*
Near or distance ⩽10 PD	33/51 (65%, CI 51 to 76%)*
Abnormal findings at day 1	
Dellen formation	1/62 (1.6%, CI 0 to 7.3%)
Corneal erosion	1/62 (1.6%, CI 0 to 7.3%)
Suspected infection	1/62 (1.6%, CI 0 to 7.3%)
Abnormal findings at month 6	
Increase in conjunctival redness	2/62 (3.2%, CI 0 to 9.8%)†

*Alternating cover test values, simultaneous angles often smaller.

†Compared with preoperative photographs.

### Postoperative management

At the end of surgery, TobraDex ointment (1 mg of dexamethasone and 3 mg of tobramycin per gram, 0.5% chlorobutanol) or Maxitrol ointment (polymyxin B sulfate 6000 units, neomycin sulfate 3500 units, dexamethasone 1.0 mg, methyl-paraben 0.05%, and propylparaben 0.01%) was applied. There was no need for an eye patch, apart from in the eye with a corneal erosion. For the first 2 weeks after surgery the following treatment was prescribed: TobraDex suspension (1 mg of dexamethasone and 3 mg of tobramycin per ml, 0.01% benzalkonium chloride) tid and TobraDex ointment in the evening or Maxitrol suspension (polymyxin B sulfate 6000 units, neomycin sulfate 3500 units, dexamethasone 1.0 mg, and benzalkonium chloride 0.004%) tid and Maxitrol ointment in the evening.

### Statistical methods

All comparisons were performed between preoperative and postoperative month 6. Binocular vision was compared using the Wilcoxon signed-rank test. Final alignment was determined with the t test. LogMAR visual acuities were analysed with the paired t test. Confidence intervals correspond to 95% the confidence level.

## RESULTS

**Figure 2:**
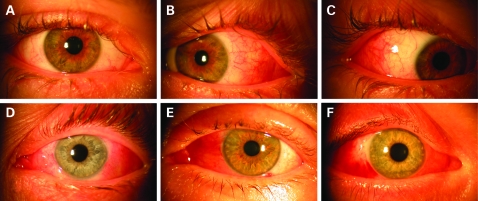
Photographs of the different types of conjunctival and lid findings 24 h after revision surgery. (A) Redness and swelling of eyelids and conjunctiva were hardly visible in the primary gaze position. (B, C) Same patient as in (A) on right and left gaze. Now, the surgical access is visible. (D) Only minimal redness or swelling of eyelids and conjunctiva in primary gaze position. (E) Moderate visibility of redness and swelling of eyelids and conjunctiva in the primary gaze position. (F) Severe visibility of conjunctival or lid swelling and redness in the primary gaze position, in this case medial subconjuctival haemorrhage and medial and lateral conjunctival chemosis*.*

Table 1 shows the preoperative characteristics of MISS patients. Fifty-one out of 56 (90.9%) consecutive patients could be included in this study. Five (9.1%) patients were lost from follow-up. None of the lost patients had an adverse outcome in the first four postoperative weeks. In 62 eyes of 51 patients (age 35.4 (16.3) years, range 4–72 years) a total of 75 horizontal rectus muscles were reoperated. On average, the patients had 2.1 strabismus surgeries previously. Twelve patients had an esotropia, two an esophoria with asthenopic complaints, 35 an exotropia and two an exophoria with asthenopic symptoms. No scleral penetration or other serious complication occurred. In one eye, the two small cuts had to be enlarged to a limbal opening in order to visualise better the operating site. This conversion was not associated with an adverse outcome. On the first postoperative day, in primary gaze position conjunctival and lid swelling and redness were hardly visible in 11 eyes (fig 2A), discrete in 15 eyes (fig 2D), moderate in 11 eyes (fig 2E), and severe in 15 eyes (fig 2F). In one patient with severe swelling and pain on eye movements, an infection was suspected, and oral antibiotics were administered. The swelling and pain resolved within 1 day. One corneal dellen and one corneal erosion occurred, which both resolved quickly. The corneal erosion was secondary to the contact of the traction suture with the cornea. The preoperative deviation at distance for esodeviations (n = 15) of 12.5 (8.5)° decreased to 2.6 (7.8)° at 6 months (p<0.001). For near, a decrease from 12.0 (10.1)° to 2.9 (1.6)° was observed (p<0.001). The preoperative deviation at distance for exodeviations (n = 35) of −16.4 (8.5)° decreased to −7.9 (6.5)° at 6 months (p<0.005). For near, a decrease from −16.5 (11.4)° to −2.9 (1.5)° was observed (p<0.005). Preoperative, best corrected logMAR visual acuity was 0.38 (0.82) compared with 0.37 (0.83) at 6 months (p>0.1). Within the first 6 months, only one patient had a reoperation. At month 6, in four patients a reoperation was planned or suggested by us because of unsatisfactory alignment. No patient experienced persistent diplopia or necessitated a reoperation because of double vision. Two patients had an increase in conjunctival redness compared with preoperatively. Stereovision improved at month 6 compared with preoperatively (p<0.01). In the majority of patients, the corneal astigmatism remained unchanged at month 6. In nine patients, the following changes were seen: 0.25 D in three patients, 0.5 D in five patients, and 0.75 D in one patient. The average dose–response relationship for distance angles at month 6 was for esodeviations 1.12° (CI 1.00 to 1.23°) and for exodeviations 1.15° (CI 1.03 to 1.27°) per millimetre of muscle displacement.

**Table 1:** Preoperative characteristics of minimally invasive strabismus surgery patients

	Frequencies
Patients	51
Eyes	62
Recessed muscles	33
Plicated muscles	15
Advanced muscles	38
Gender male/female	21/30 (41%)
Age (years)	35.4 (SD 16.3)

## DISCUSSION

Minimally invasive techniques are becoming important in almost every field of surgery including ophthalmic surgery. Instrument miniaturisation, endoillumination and optical improvements have changed and will continue to strongly influence the way in which surgery is performed. In this study, the results of horizontal rectus muscles revision surgeries with a novel MISS technique in 51 patients have been presented. Squint surgery is performed through two small radial cuts along the superior and inferior muscle margin (fig 1). Despite restricted openings, scarring surrounding the insertions could be adequately detached and, if necessary, resected. Since patients with previous posterior fixation sutures were excluded in this patient series, no patient harboured excessive posterior scarring. For scars lying more behind, a considerable enlargement of the small cuts is necessary. Postoperatively, these openings remain covered by the eyelids apart from during excessive upgaze and excessive lateral gaze, which postoperatively minimises visibility of the surgical procedure. If a better visibility of the operative site is necessary, this type of cut can be prolonged anteriorly or even joined with a limbal cut (fig 1O). In this patient series, this was necessary for one eye. Conversion was not associated with an adverse course. The whole surgical procedure can be performed with the same instruments used for usual limbal approach. There is no need for an assistant. Despite a strongly restricted opening, the MISS technique allowed adequate muscle exposure to perform recessions, plications and advancements, thus minimising anatomical disruption. Two to 3 weeks after surgery, the eyes often looked normal or nearly normal in the primary gaze position. In a few eyes, this was already achieved on the first postoperative day. A conjunctival opening situated far away from the cornea should decrease the incidence of corneal dellen formation, avoid a prolapse of the Tenon capsule, and minimise postoperative discomfort. There is also increasing evidence that non-limbal strabismus surgery affects less perilimbal blood supply and may safeguard against anterior-segment ischaemia in high-risk patients. However, because of the low incidence of such complications, only larger studies will be able to show if such complications will be less frequent with the new technique. MISS revision surgery also avoids further traumatising of already scarred perilimbal conjunctiva and, in such cases, considerably shortens operating times. Although not objectively measured, we had the impression that in the immediate postoperative period, patient discomfort was reduced. This is supported by the fact that only one patient needed eye patching after surgery. At 6 months, only a minimal scarring was found along the incision lines, and only in two patients was an increase in conjunctival redness observed. It could be assumed that this minimal cicatrisation might facilitate further reoperations.

Parks’s fornix opening^14^ also avoids corneal complications. The advantage of Parks’s technique is the better visualisation of the surgical site, while MISS can be performed without an assistant and also in older patients with inelastic conjunctiva.

Although these results are promising, definitive superiority of MISS over the traditional, limbal approach or Parks fornix opening has to be proven by other reports, since increased incidences of rare complications could have been missed in this study—for example the frequency of endophthalmitis.^19^

In summary, the results of a new surgical technique for horizontal rectus muscle surgery have been presented, which seems to be safe and more rational than previous openings. Incision placement where the surgical procedure of the muscle occurs allows one to minimise the total opening size, to reduce postoperative discomfort and possibly also to reduce hospital stay, working disability and complications related to limbal approaches.
